# Cerebrospinal fluid absorption block at the vertex in chronic hydrocephalus: obstructed arachnoid granulations or elevated venous pressure?

**DOI:** 10.1186/2045-8118-11-11

**Published:** 2014-05-23

**Authors:** Grant A Bateman, Sabbir H Siddique

**Affiliations:** 1 Department of Medical Imaging, John Hunter Hospital, Locked Bag 1, Newcastle Region Mail Center, Newcastle 2310, Australia; 2 Newcastle University Faculty of Health, Callaghan Campus, Newcastle, Australia

**Keywords:** Chronic hydrocephalus, Normal pressure hydrocephalus, MR venography, Cerebral blood flow, Sagittal sinus pressure

## Abstract

**Background:**

The lack of absorption of CSF at the vertex in chronic hydrocephalus has been ascribed to an elevation in the arachnoid granulation outflow resistance (R_out_). The CSF infusion studies measuring R_out_ are dependent on venous sinus pressure but little is known about the changes in pressure which occur throughout life or with the development of hydrocephalus.

**Methods:**

Twenty patients with chronic hydrocephalus underwent MR venography and MR flow quantification techniques. The venous outflow pressure was estimated from the sinus blood flow and the cross-sectional area of the transverse sinuses. Adult controls as well as a normal young cohort were selected to estimate the change in sinus pressure which occurs throughout life and following the development of hydrocephalus. Significance was tested with a Student’s t-test.

**Results:**

The size of the transverse sinuses was unchanged from the 1^st^ to the 5^th^ decade of life, indicating a stable outflow resistance. However, the blood flow was reduced by 42%, indicating a likely similar reduction in pressure gradient across the sinuses. The sinuses of hydrocephalus patients were 38% smaller than matched controls, indicating a 2.5 times increase in resistance. Despite the 24% reduction in blood flow, a significant increase in sinus pressure is suggested.

**Conclusions:**

The size of the venous sinuses normally does not change over the age range investigated but sinus pressure is reduced proportional to an age-related blood flow reduction. Hydrocephalus is associated with much smaller sinuses than normal and an elevation in venous pressure may explain the lack of CSF absorption into the arachnoid granulations in chronic hydrocephalus.

## Background

It is known that all forms of communicating hydrocephalus are associated with a reduction in CSF flow to the vertex, with the fluid refluxing into the ventricles instead [1]. This phenomenon has been suggested to be evidence for a blockage of CSF flow at the vertex. Davson *et al.* modelled the CSF absorption over the vertex through the arachnoid granulations and into the venous sinuses. He suggested that the intracranial pressure (ICP) should be dependent on the CSF formation rate (FR_CSF_), the resistance to flow through the arachnoid granulations (R_out_) and the sagittal sinus pressure (P_SSS_). The equation ICP = R_out_ × FR_CSF_ + P_SSS_ was derived to explain this relationship [2]. Ekstedt studied CSF absorption utilizing a mock CSF infusion method. He found the CSF pressure, CSF outflow resistance, CSF formation rate and sagittal sinus pressure did not vary with age, with the sagittal sinus pressure being 7.5 mmHg on average [3]. He went on to show that the reduction in CSF absorption over the vertex in chronic hydrocephalus was due to a significant increase in the CSF outflow resistance [3]. This has remained the accepted explanation for the apparent block to CSF flow at the vertex since. It is assumed that the CSF formation rate and sagittal sinus pressures are constants during the test. Under these constraints, if the CSF formation rate is artificially increased by infusion of mock CSF at known rates, and the resultant ICP is measured, then the slope of the line generated when pressure is plotted against flow rate will be the outflow resistance.

The test is heavily dependent on the sinus pressure remaining constant during the course of the study. For example, in a modeling study using 2-day old rats it was found that the sinus walls were more elastic and deformable than in the adult animals. When an infusion study was performed, the venous pressure rose in the younger rats during the test compared to the adults where it was constant. Ignoring the rise in venous pressure overestimated the resistance to CSF flow across the sinus wall fivefold in the juvenile rats compared to when the venous pressures were taken into account. In adult rats, the infusion study was accurate because the venous pressure was a constant [4]. Similarly, it has recently been shown in patients with pseudotumor cerebri and slit ventricle syndrome that the venous pressure does rise during the infusion study and therefore the CSF outflow resistance is overestimated approximately 5 fold in these disorders as well [5]. Could an overestimation of R_out_ also occur in chronic hydrocephalus? The venous pressure is not routinely measured in an infusion study and has been rarely measured in normal controls due to the invasive nature of retrograde manometry. Ekstedt’s finding that the sinus pressure does not change throughout life is also problematic given that the blood flow through the sagittal sinus varies significantly throughout life. At 10 yrs the average flow is approximately 600 ml/min, at 45 years it is 400 ml/min and at 80 years about 250 ml/min [6]. Using Ohm’s law, where the pressure is dependent on the flow and outflow resistance, maintaining a constant sinus pressure throughout life would require a significant reduction in the cross-sectional area of the sinuses. Whilst we cannot directly study the sinus pressure in normal aging or during hydrocephalus due to the ethical constraints from the invasive nature of manometry, we can measure the blood flow through the sinuses and their cross-sectional areas using non-invasive MRI techniques. Using Poiseuille’s law, sinus pressure can be estimated if the constants in the equation can be calculated. We can calibrate the calculation of sinus pressure and obtain a figure for the constants in Poiseuille’s law by utilizing the limited available literature on normal sinus pressure at manometry and use the derived equation to study the changes in sinus pressure across normal aging and secondary to chronic hydrocephalus. Thus, the purpose of this study is to measure the venous sinus outflow blood volume and the sinus cross-sectional area in a cohort of individuals with chronic hydrocephalus and compare the estimated sinus pressures with those of a group of age-matched controls and a group of young healthy individuals.

## Methods

### Subjects

Patients referred for investigation of chronic hydrocephalus have been routinely studied with MRI at the John Hunter Hospital, Newcastle Australia, from July 2011 to October 2013. As part of the standard protocol, MRV and MR flow quantification studies have been acquired. Twenty patients were enrolled in the study; there were 7 females and 13 males. The mean age was 45 ± 10 years. Patients younger than 30 years were excluded to ensure some chronicity of the disease process and those over 65 years were excluded to reduce the likelihood of comorbidity from dementia or atrophy. Patients with chronic idiopathic communicating hydrocephalus without a currently functioning shunt were selected. Eight patients fulfilled the clinical criteria for probable normal pressure hydrocephalus and had received no prior therapy, six patients had hydrocephalus found incidentally on imaging and could be given the diagnosis of LOVA (late onset ventriculomegaly of adults), four were found to have hydrocephalus following investigation of headaches but without gait disturbance and in two there was hydrocephalus presenting with a failed shunt tube confirmed at nuclear cisternography. The controls were selected from a bank of normal patients and volunteers acquired from previously published material [7]–[9]. The controls were selected from consecutive patients undergoing MRI examinations for indications unrelated to headaches or CSF flow abnormalities where the MRI examination was seen to be without structural abnormality. The normal young patients averaged 10 ± 4 years with 5 males and 5 females. The normal adults were selected to match the hydrocephalus patients with a mean age 44 ± 10 years with 8 females and 12 males. There was no clinical suspicion of raised intracranial pressure or significant history of headache in these individuals. The protocol was approved by the Hospital ethics committee and written informed consent was obtained from each patient.

### MR and analysis

All patients were imaged on a 1.5 T superconducting magnet (Vario; Seimens, Erlangen Germany). The patients were scanned with standard T1 sagittal, T2 and FLAIR axial images as well as a standard 2D time of flight MR venogram sequence acquired in a slightly oblique sagittal plane. The MR flow quantification sequence was acquired as a phase contrast study with retrospective cardiac gating. The TR was 26.5 msec, TE 6.9 msec, flip angle 15°, slice thickness 5 mm, matrix 192 × 512, FOV 150 and a single excitation. The velocity encoding value was 40 cm/sec. The plane was selected to pass through the sagittal sinus 2 cm above the torcular and through the mid part of the straight sinus. The planar imaging, as well as the flow quantification raw data, was archived on a hard drive.

The Evan’s index was calculated for the hydrocephalus patients with the cross-sectional width of the ventricles being divided by the width of the anterior cranial fossa from inner table to inner table along a line in the same position as the ventricles. The MRV was reconstructed into 5 mm sagittal slices, with a slice from the right and left half mid-way from the center line to the inner table of the skull selected. At this site, the mid portion of the transverse sinus is in cross-section and the area of the lumen of each sinus was measured by tracing the outline of the sinus using the proprietary measuring tool. The right and left sinuses were added together to obtain the total outflow area. Using the flow data, regions of interest were placed around the sagittal and straight sinuses in each patient. Care was taken to exclude aliasing by retrospectively manipulating the base lines of each resultant graph. Background subtraction was utilized. The addition of the flow from the two sinuses gave the total outflow blood volume. The mean values and standard deviations were calculated for each measurement. The significance of the findings when hydrocephalus was compared with the normal adults was tested using a Student’s t-test with a *p* value less than 0.05 used to indicate significance. The sagittal sinus pressure was estimated for each patient using a modified Poiseuille equation.

### Theory behind sagittal sinus pressure estimation

The pressure in the sagittal sinus in the supine position is dependent on the jugular bulb pressure and the pressure drop which occurs across the sinuses. The jugular bulb pressure is essentially equal to the central venous pressure due to the capacious nature of the jugular veins. There is no significant change in central pressure throughout life [10]. In children 6–14 years the average central venous pressure is 6 mmHg in the supine position [11]. The mean central venous pressure in adults is 5 ± 0.7 mmHg in the supine position [12]. The pressure drop across a vessel is calculated using Poiseuille’s equation:

(1) $$\Delta P=8µ\mathrm{LQ}/\pi r^{4}$$

Where ΔP is the pressure drop, μ is the viscosity, L is the length of the vessel, Q is the fluid flow rate, π is the proportionality constant relating the diameter to the circumference of a circle and r is the radius of the vessel. The cross-sectional area (A) of a vessel is given by the equation:

(2) $$A=\pi r^{2}$$

By squaring both sides we get:

(3) $$A^{2}=\pi^{2}r^{4}$$

By taking equation 1 and multiplying both the numerator and denominator by π we get:

(4) $$\Delta P=8µ\;\pi \;\mathrm{LQ}/\pi^{2}r^{4}$$

By using equation 3 we can substitute A^2^ for the denominator in equation 4:

(5) $$\Delta P=8µ\;\pi \;\mathrm{LQ}/A^{2}$$

As the venous sinuses are lined by dura which is attached to bone the length of these sinuses does not change. Similarly, the viscosity of the blood and π are constants so equation 5 can be simplified to:

(6) $$\Delta P=\mathrm{kQ}/A^{2}$$

We can find the value of k by using known values from the literature. Grady *et al.* measured the sagittal sinus pressure at manometry in 15 children ranging in ages from 1–17 yr and found the average pressure to be 10 mmHg in the supine position [13]. Iwabuchi *et al.* measured the sagittal sinus pressure in 11 children mean age 7 yr and found the pressure to be between 10 and 13 mmHg [14]. Pooling the data gives an average pressure of 10.5 mmHg. Subtracting the central venous pressure for children (6 mmHg) this gives a pressure drop of 4.5 mmHg across the sinuses. The data from the current study gives the flow rate through the sinuses to be 810 ml/min and the area of the sinuses to be 73 mm^2^ for the children studied. Therefore the constant k in formula 6 can be calculated to be, 4.5 × (73)^2^/810 = 29.6. Thus the sagittal sinus pressure can be estimated to be the pressure drop (i.e. equation 6) plus the central venous pressure:

(7) $$P_{\text{SSS}}=29.6Q/A^{2}+\mathrm{CVP}$$

Equation 7 was utilised to estimate the sinus pressures for each group of patients with the findings presented in Table 1.

**Table 1:** Sinus blood flow and estimated sinus pressure in normal young and adult patients and in hydrocephalic patients

**Group**	**Age**	**SSS outflow**	**ST outflow**	**Total outflow**	**Estimated sinus pressure**
	**Yr**	**ml/min**	**ml/min**	**ml/min**	**mmHg**
Normal young n = 10					
Mean	10	620	190	810	10.5
SD	4	160	60	220	3.6
Normal adult n = 20					
Mean	44	360	110	470	7.7
SD	10	80	40	110	1.1
Hydrocephalus n = 20					
Mean	45	280	75	355	10.2
SD	10	60	40	80	5.5
p value	0.81	0.002*	0.01*	0.001*	0.008*

Mean +/- standard deviation; SSS: superior sagittal sinus, ST: straight sinus, *: t-test *p* value < 0.05 normal adults versus hydrocephalic patients.

## Results

The transverse sinus area data is summarized in Table 2, with the blood flow and estimated venous pressure data summarized in Table 1. There was no significant difference between the cross-sectional area of the normal young and normal adult groups. The ventricles of the hydrocephalus patients were enlarged, with an Evan’s index of 0.47 ± 0.07 and the controls all being less than 0.3. The cross-sectional area of the transverse sinus in the hydrocephalus patients was a 38% smaller compared to the normal adults (*p* = 0.0001), with the majority of this reduction occurring on the right side. On average the right transverse sinus represented 59% ± 22%of the total sinus area in the controls and 55% ±22% in the adult hydrocephalus patients (Table 2). As expected, there was a lower sinus blood flow in the normal adults compared to the normal young, and the estimated sinus pressure was reduced proportional to the blood flow. The venous sinus blood flow in the hydrocephalus patients was reduced by 24% compared with the normal adults (*p* = 0.001). The estimated out flow pressure in the hydrocephalus patients was 32% larger than the normal adults (*p* = 0.008, Table 1).

**Table 2:** Cross-sectional area of the transverse sinuses measured by MR imaging in normal young and adult patients and in hydrocephalic patients

**Group**	**Age**	**Right TS**	**Left TS**	**Total area**
	**Yr**	**mm**^**2**^	**mm**^**2**^	**mm**^**2**^
Normal young n = 10				
Mean	10	47	27	73
SD	4	15	19	26
Normal adult n = 20				
mean	44	43	29	72
SD	10	19	15	16
Hydrocephalus n = 20				
mean	45	25	20	45
SD	10	11	10	15
*p* value	0.81	0.001*	0.04*	0.0001*

Values are means +/- standard deviation, TS, transverse sinus; *: t-test *p* value < 0.05 normal adults versus hydrocephalus patients.

## Illustrative case

**Figure 1:**
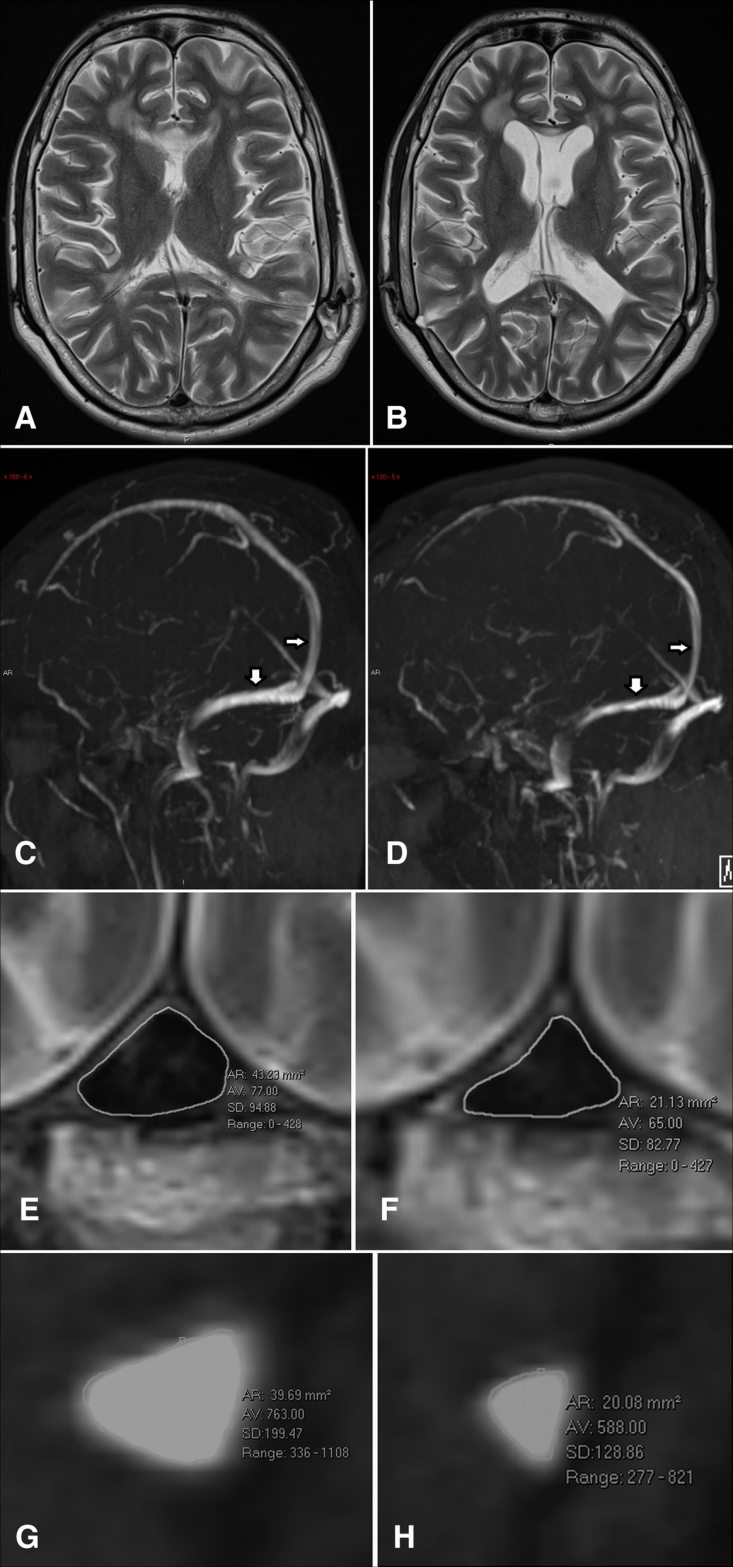
**MRI imaging of a chronic hydrocephalus patient before and after shunt failure. A** A T2 axial image of a 32 year-old male with chronic hydrocephalus and a left occipital shunt currently functioning. **B** A T2 axial image following removal of the shunt showing enlargement of the ventricles. **C** The MR venogram with functioning shunt appears normal, the thin arrow indicates the sagittal sinus and the thick the dominant transverse sinus. **D** The MR venogram with the shunt removed shows the sagittal and transverse sinuses to be smaller than previously. This is most easily seen at the level of the arrows. **E** A T2 axial image of the sagittal sinus 2 cm above the torcular taken at the level of the thin arrows in **C** and **D**) showing the cross-sectional area of the lumen to be 43 mm^2^. **F** The follow-up T2 axial image at the same level as 1e but with shunt removed shows the lumen to be 21 mm^2^. **G** A sagittal reconstruction of the mid portion of the right transverse sinus (at approximately the level of the thick arrows above) taken from the MRV raw data shows the sinus to be 40 mm^2^ in area. **H** The same reconstruction as 1 g following shunt removal shows the sinus area to be 20 mm^2^.

This 32 yr old male had communicating hydrocephalus diagnosed as a child and had required many shunt revisions over the years. His most recent revision was in July 2013 where a Medtronic medium pressure valve with an opening pressure of about 7 mmHg was inserted. A baseline MRI study was performed and this confirmed a normal ventricular size (Figure 1A). Three months later following another shunt obstruction, the shunt was removed and a Rickham’s reservoir inserted to gauge shunt dependence. A follow-up MRI in October confirmed the ventricular enlargement (Figure 1B). Whilst the shunt was working, the venous sinuses appeared normal (Figure 1C), with the total cross-sectional area of the transverse sinuses being 61 mm^2^, which was comparable to the controls. The venous outflow volume was 369 ml/min. The estimated pressure in the sagittal sinus was 7.9 mmHg based on the sinus area and the volume of flow. Figure 1D is the appearances of the sinuses after shunt removal and all the sinuses appear slightly smaller. The projection images are misleading as to the degree of this change, however, the total transverse sinus area was much less than previously noted at 35 mm^2^. The blood flow had also reduced at 245 ml/min. The estimated sagittal sinus pressure was 10.9 mmHg. At this point, retrograde manometry was performed to check the venous pressure and exclude a focal stenosis, which could be a target for treatment. The manometry confirmed the pressure at the base of the sagittal sinus to be 11 mmHg compared to atmospheric pressure at the external auditory meatus in the supine position. No focal stenosis was found. At this time, overnight manometry using the reservoir confirmed the CSF pressure to be 13–14 mmHg. Using a modified Masserman technique the drain pressure was lowered to 3 mmHg below the opening pressure and the CSF collected for 24 h. The estimated CSF formation rate was 0.22 ml/min. The 50% change in cross-sectional area of the sagittal sinus was confirmed on T2 images taken 2 cm above the Torcular (Figure 1E, F) and the 50% reduction in right transverse sinus area is shown in Figures 1g and 1h.

## Discussion

The literature surrounding the investigation of chronic hydrocephalus abounds with papers utilising various forms of infusion or bolus injection to discover the out flow resistance or R_out_. All of these studies are based on Davson’s equation, which as previously discussed is in the form:

$$\mathrm{ICP}=R_{\text{out}}\times {\mathrm{FR}}_{\text{CSF}}+P_{\text{SSS}}$$

We can see that the sagittal sinus pressure is an important element in the calculation of R_out_ but the sinus pressure is almost never measured. It is either calculated from Davson’s equation itself (circular reasoning) or assumed to be a constant. Calculations of the sagittal sinus pressure by Ekstedt suggested that the sinus pressure does not change throughout life [3] but as already discussed, this would appear to be unlikely. The purpose of the current study was to estimate the sinus pressure using a non-invasive technique based on a modified Poiseuille equation, which is therefore independent of Davson’s equation. This will be used to test how well infusion studies perform throughout life and following the development of communicating hydrocephalus.

The current study utilised a modification of Poiseuille’s equation using vascular area and blood flow data obtained from the current study. The proportionality constant in the equation was derived from manometry pressure measurements in children obtained from the literature (see methods). The mean sinus pressure for normal children obtained from the literature was 10.5 mmHg. The derived equation was then used to predict the sinus pressures in a normal adult cohort independent of the literature. A mean value of 7.7 mmHg was obtained. This compares well to Ekstedt’s reference value for the sinus pressure of 7.5 mmHg [3]. Martins *et al.* measured the sagittal sinus pressure in adults aged 18 to 60 yr and found that in the 9 individuals where the CSF pressure was independent of the ICP, the sinus pressure averaged 8.0 mmHg [15] which again is very similar to the figure predicted by the current study. Iwabuchi *et al.* found the sinus pressure to be between 4.8 and 9.1 mmHg in adults of mean age 45 years, the range depending on the technique used [14]. Therefore, the current technique appears to correlate well with the available literature for adults indicating an acceptable precision. These findings indicate that the sinus pressure is reduced with age. The cross-sectional area of the sinuses does not appear to change from the first decade to the fifth, indicating the venous outflow resistance is unchanged throughout this period of normal life. The reduction in pressure appears to be directly proportional to the reduction in blood flow.

### Davson’s equation revisited

Having established a normal range for sinus pressures, we can test Davson’s equation by utilising the most up to date figures available in the literature for the ICP, R_out_ and FR_CSF_ and calculate the sinus pressure using this technique. In a large study, the average CSF pressure in 10-year old children was found to be 14.6 mmHg [16]. The R_out_ has been found to depend linearly with age, with the regression line being; 9.88 + 0.075 × Age in mmHg/ml/min [17]. This gives a R_out_ of 10.63 mmHg/ml/min at 10 years. The CSF formation rate is highest in children and young adults, being about 0.4 ml/min and decreases with age to about 50% of this value at age 70 years [18]. Therefore, Davson’s equation predicts an average sinus pressure of 10.3 mmHg (14.6- 10.63× 0.4) in children with a pressure gradient across the arachnoid granulations of 4.3 mmHg for CSF to flow. This sinus pressure correlates well with the literature (10.5 mmHg). Similarly, in normal middle age, the CSF pressure in a very large study averaged 11.5 mmHg at 45 years [19]. Using the equation as discussed above, the R_out_ at 45 years would be 13.3 mmHg/ml/min. The CSF formation rate is reduced by 50% in old age, and its reduction is said to be linear throughout life [20]. Therefore, we can estimate a 25% reduction in middle age giving a figure of 0.3 ml/min. Thus, Davson’s equation estimates the sinus pressure to be 7.5 mmHg (11.5- 13.3× 0.3) with a pressure gradient of 4 mmHg across the arachnoid granulations in middle age. This sinus pressure is similar to the published literature, and the findings in the current study (7.7 mmHg), indicating that CSF infusion studies are probably quite accurate in normal individuals.

In patients with chronic hydrocephalus of average age 54 years, the average CSF pressure was found to be 1.5 mmHg higher than the control group [20], which would give a CSF pressure of 13 mmHg at 45 years. The average CSF formation rate in the hydrocephalus patients was 0.25 ml/min [20]. In the Dutch normal-pressure hydrocephalus study, a good outcome from treatment of NPH was found to be in individuals with a R_out_ greater than 18 mmHg/ml/min but averaging approximately 24 mmHg/ml/min [21]. Similarly, Czosnyka *et al.* noted that the upper limit of normal for R_out_ is about 12 mmHg/ml/min, with NPH patients often being twice this amount [22]. Thus, the R_out_ in chronic hydrocephalus is about 24 mmHg/ml/min. Therefore, Davson’s equation estimates the venous pressure to be 7 mmHg (13-24× 0.25) in chronic hydrocephalus with a pressure gradient across the arachnoid granulations of 6 mmHg. We can see there is a discrepancy between the current study and infusion studies in hydrocephalus. Infusion studies predict a normal sagittal sinus pressure in hydrocephalus (7 mmHg) but the estimation of the sinus pressure based on flow and area of the sinuses suggests 10.2 mmHg or 3.2 mmHg higher. Which figure is correct? There is almost no information in the literature regarding sinus pressure in chronic hydrocephalus. Hash *et al.* noted that an attempt to shunt CSF directly into the sagittal sinus in a patient with NPH failed because the sinus pressure was 1 mmHg higher than the CSF pressure (i.e. elevated) and there was no pressure gradient for CSF absorption [23]. In another study, a prediction of an elevation in sinus pressure of 3–4 mmHg above normal in chronic hydrocephalus was made based on evidence of increased collateral flow bypassing the sinuses [24]. In a kaolin dog model of chronic hydrocephalus, the initial phase was associated with an elevation in CSF and sinus venous pressure, with a normal CSF to sagittal sinus gradient. In the chronic phase, the CSF pressure returned to normal and there was some reduction in sinus pressure but it remained elevated with loss of the pressure gradient across the arachnoid granulations [25]. Similarly, in a rat model of hydrocephalus, there was loss of the pressure gradient between the CSF and sinus during infusion studies with the venous pressure rising linearly with CSF pressure [26]. The illustrative case in the current study appears similar to this literature. Without a shunt, the sinus pressure was 11 mmHg and the CSF pressure 13–14 mmHg, giving a gradient across the arachnoid granulations of 2–3 mmHg. We know from the predictions of Davson’s equation that in normal middle aged subjects, a gradient pressure of about 4 mmHg is required for CSF to flow. Therefore, the lack of absorption at the vertex appears to be due to an unfavourable pressure gradient and not blocked granulations in this case (in the later instance the gradient pressure should have been increased). Similarly, the pooled data suggests a CSF pressure of 13 mmHg in chronic hydrocephalus with a sinus pressure of 10.2 mmHg, giving a gradient across the granulations of 2.8 mmHg and therefore no CSF flow.

The current study would tend to suggest that infusion studies underestimate the venous pressure in hydrocephalus. If we corrected Davson’s equation for a sinus pressure of 10.2 mmHg, then in order for the equation to balance, either the R_out_ or the CSF formation rate must have been over estimated. The estimate of the CSF formation rate is made by reducing the CSF pressure significantly and measuring the CSF flow required to maintain this pressure. It is said the CSF formation rate is not altered by CSF pressure, so it is unlikely to be overestimated [27]. In the illustrative case the CSF formation rate was 0.22 ml/min which compares well with the literature [20]. Thus, the R_out_ is probably at fault. The R_out_ corrected for a sinus pressure of 10.2 mmHg would average 11.2 mmHg/min/min ((13–10.2)/0.25) for the hydrocephalus cohort in order to balance Davson’s equation. Thus, if the sinus pressure figure of 10.2 mmHg is correct, then the R_out_ in chronic hydrocephalus is actually normal. Therefore, it is being overestimated two fold by infusion studies. In the illustrative case, the ICP whilst the ventricular drain was monitored averaged 13.5 mmHg, the formation rate was 0.22 ml/min and the sinus pressure was 11 mmHg. Therefore Davson’s equation gives the actual R_out_ to be (13.5-11)/ 0.22 = 11.4 mmHg/ml/min in this case which is normal and similar to the pooled data figure just discussed.

### A cause for R_out_ overestimation

In a recent study, R_out_ was found to be overestimated if the venous pressure increased during the course of the infusion study. The degree of this overestimation was dependent on the proportion of the CSF pressure which was fed back to the sinuses. In pseudotumor cerebri, if 80% of the increase in CSF pressure occurring during the study was fed back to the sinuses then the R_out_ was overestimated 5 fold i.e. if the CSF pressure was raised by 10 mmHg during the test and collapse of the sinuses allowed them to increase in pressure by 8 mmHg, then the test would overestimate a normal R_out_ as being elevated five times normal [5]. The two fold overestimation found in the current study could be accounted for by a feedback percentage of 50%. In the illustrative case, whilst the shunt was working the CSF pressure was set by the valve at about 7 mmHg and the estimate of the sinus pressure was 7.9 mmHg. When the shunt was removed, the CSF pressure went up to about 13.5 mmHg or an increase of 6.5 mmHg. The sinus pressure went up to 11 mmHg or an increase in pressure of 3.1 mmHg. Therefore, the increase in CSF pressure increased the sinus pressure by passive collapse. The feedback percentage was 50%. Thus, if an infusion study were performed it would overestimate the R_out_, in this case two fold due to the collapse of the sinuses (i.e. to about 22.8 mmHg/ml/min).

How wide spread is this problem? Obviously if infusion studies are accurate in normal patients, then the sinuses of normal patients do not collapse to any significant degree. In a study where ICP was altered by the addition or removal of CSF, nine of twelve patients showed no change in sinus pressure, despite the CSF pressure being raised by up to 75 mmHg. Therefore, there was no venous collapse in these cases and an infusion study would be accurate. In the remaining three patients the sagittal sinus pressure increased by 12 mmHg during a 20 mmHg elevation in CSF pressure (about a 60% feedback fraction). In one of these patients, a venogram showed a partial collapse of both the sagittal and transverse sinuses during the raised CSF pressure [15] (similar to the illustrative case). If an infusion study were performed on these three individuals it would overestimate R_out_ by over two fold.

### The underlying pathophysiology of chronic hydrocephalus

If the venous pressures rise by 3 mmHg in chronic hydrocephalus, why do the CSF pressures increase by only 1.5 mmHg [20]? A moderation of the CSF pressure would require a parallel CSF outflow pathway, other than the arachnoid granulations, to reduce the total outflow resistance and make up for the unfavourable gradient pressure across the granulations. We know there is transependymal CSF absorption in hydrocephalus [28] and this may provide a parallel pathway. It has been suggested that capillary absorption is not possible because the CSF pressure would need to be above the capillary pressure and the capillaries would collapse [29]. However, the absorption of water across a capillary bed depends on all the Starling forces not just the hydrostatic pressure. It has been estimated that no net absorption or filtration of water would occur with an average capillary bed pressure of 32 mmHg in the brain [30]. If the capillary bed were reduced from 32 mmHg to anywhere above 13 mmHg, then the capillaries would absorb water but maintain their blood flow at a lower level. Below 13 mmHg the capillaries would start to collapse and blood flow would cease. In chronic hydrocephalus the subependymal white matter is ischemic [31,32]. Therefore, there is a reduction in blood flow at a reduced capillary pressure, bringing about bulk water absorption and moderating the CSF pressure.

If the venous pressure is raised why don’t all patients have small ventricles like pseudotumor cerebri? Whether or not the ventricles dilate depends on brain turgor. If the brain is stiff the ventricles will not dilate, if it is more compliant they will. Brain turgor is predominantly affected by the medullary venous pressure [5]. If the sinuses collapse during an elevation in CSF pressure and 80-90% of the CSF pressure is fed back to the veins, then the medullary veins will be close to CSF pressure and no ventricular dilatation will ensue i.e. pseudotumor cerebri or slit ventricle syndrome [5]. If the feedback fraction is 50% then the venous pressure will lag behind the CSF pressure. Also, the subependymal white matter is ischemic. Therefore, the medullary pressure is lower, so brain turgor is less in this region, and the ventricles may enlarge [5].

### Study limitations

The present study limits its scope to patients between the ages of 30 and 65 years because of the risk of significant co-morbidity from dementia and atrophy in older patients. Therefore, the applicability to NPH patients in the age group over 65 may be limited until further research is done. The methods utilise MRI, which requires quiet respiration in a supine patient, so it is difficult to draw conclusions as to how the sinuses may react to the upright posture or valsalva manoeuvre. These limitations are common to most hydrocephalus research. The central venous pressures were not measured directly but estimated to be normal, given the patients were not morbidly obese or in right heart failure, this is probably justified.

Poiseuille’s equation assumes laminar flow in a uniform cylinder with rigid smooth walls. It is obvious that the sinuses have bends in the sigmoid region, there is some irregularity to the walls and probably wall movement. Thus, the calculations can only be a first approximation to reality. The flow is probably laminar in the sinuses due to the low Reynold’s numbers involved. The wall irregularity and bends would be similar between the controls and test patients but the wall pulsation is probably greater in the more compliant sinus walls in the hydrocephalus patients. The flexible walls of the sinus distort in the hydrocephalus patients and the sinuses become more triangular and less cylindrical compared to the controls. As triangular pipes are less efficient, the effect may have been to underestimate the resistance slightly in the hydrocephalus group compared to the controls.

The outflow resistance of both transverse sinuses was added together and both sinuses assumed to act as a single resistor because this considerably simplified the calculations. This would be a reasonable assumption if the ratio of the resistances between the right and left sinuses remained the same in the control and test groups. If there was a significant variation, then depending on the magnitude, the pressure would be over or under estimated. The ratio of the average right and left transverse sinus resistances for the adult controls was 2.2:1 and for the hydrocephalus patients 1.56:1. Recalculating the pressure gradient across the sinuses in the hydrocephalus patients taking the parallel resistances into account provided an estimate of 9.95 mmHg compared to the quoted figure of 10.2 mmHg or a 2.5% error which did not affect the outcome of the study.

## Conclusions

The size of the cerebral venous sinuses normally does not change from the first to the fifth decades of life but there is a reduction in blood flow through the sinuses and sinus pressure is proportional to the age-related blood flow reduction. Chronic hydrocephalus is associated with much smaller sinuses than normal and despite some reduction in blood flow, there is an elevation in venous pressure. This may explain the lack of CSF absorption into the arachnoid granulations because the pressure gradient across the granulations is not favourable in chronic hydrocephalus. An increase in subependymal CSF absorption probably moderates any increase in CSF pressure which would have otherwise occurred.

## Abbreviations

ICP: Intracranial pressure; FR_(CSF)_: Cerebrospinal fluid formation rate; P_sss_: Pressure in the sagittal sinus; R_out_: Cerebrospinal fluid outflow resistance.

## Competing interests

The authors declare we have no conflict of interest concerning the materials or methods used in this study or the findings specified in this paper. There are no competing interests.

## Authors’ contributions

GB conceived and designed the study, obtained and processed the data, performed the statistical analysis and wrote the manuscript. SS was involved in patient selection, consent and data acquisition. Both authors have read and approved the final version of the manuscript.

## Authors’ information

GB is a Neuroradiologist and currently Director or MRI at John Hunter Hospital in Newcastle, Australia. He received a doctorate degree from the University of Sydney for a thesis based on the MRI investigation of CSF disorders. He is a conjoint associate professor at the University of Newcastle in the faculty of health.

SS is a consultant radiologist at John Hunter Hospital and a conjoint lecturer at the University of Newcastle.
